# A *Bacillus velezensis* strain shows antimicrobial activity against soilborne and foliar fungi and oomycetes

**DOI:** 10.3389/ffunb.2024.1332755

**Published:** 2024-02-23

**Authors:** Anna Wockenfuss, Kevin Chan, Jessica G. Cooper, Timothy Chaya, Megan A. Mauriello, Sarah M. Yannarell, Julia A. Maresca, Nicole M. Donofrio

**Affiliations:** ^1^ Microbiology Graduate Program, University of Delaware, Newark, DE, United States; ^2^ Department of Plant and Soil Sciences, University of Delaware, Newark, DE, United States; ^3^ Department of Chemistry and Biochemistry, University of Delaware, Newark, DE, United States; ^4^ Department of Civil and Environmental Engineering, University of Delaware, Newark, DE, United States

**Keywords:** biocontrol agent, antifungals, Magnaporthe oryzae, appressorial formation, plant pathogens, hyphae

## Abstract

Biological control uses naturally occurring antagonists such as bacteria or fungi for environmentally friendly control of plant pathogens. *Bacillus* spp. have been used for biocontrol of numerous plant and insect pests and are well-known to synthesize a variety of bioactive secondary metabolites. We hypothesized that bacteria isolated from agricultural soil would be effective antagonists of soilborne fungal pathogens. Here, we show that the Delaware soil isolate *Bacillus velezensis* strain S4 has *in vitro* activity against soilborne and foliar plant pathogenic fungi, including two with a large host range, and one oomycete. Further, this strain shows putative protease and cellulase activity, consistent with our prior finding that the genome of this organism is highly enriched in antifungal and antimicrobial biosynthetic gene clusters. We demonstrate that this bacterium causes changes to the fungal and oomycete hyphae at the inhibition zone, with some of the hyphae forming bubble-like structures and irregular branching. We tested strain S4 against *Magnaporthe oryzae* spores, which typically form germ tubes and penetration structures called appressoria, on the surface of the leaf. Our results suggest that after 12 hours of incubation with the bacterium, fungal spores form germ tubes, but instead of producing appressoria, they appear to form rounded, bubble-like structures. Future work will investigate whether a single antifungal molecule induces all these effects, or if they are the result of a combination of bacterially produced antimicrobials.

## 1 Introduction

A secure food supply relies heavily on managing pathogens and pests that plague major crops. Plant diseases result in a loss of $220 billion/year, according to the Food and Agricultural Organization (Food and Agricultural Organization, 2021), and a recent study demonstrated that as temperatures rise, soilborne fungal plant pathogens will increase in worldwide abundance (Delgado-Baquerizo et al., 2020). A global survey of wheat, soybean, rice, potato and maize indicates between 17 to 30% of these crops will be lost due to plant disease and up to 20% of crops can be lost to fungal diseases post-harvest (Fisher et al., 2012; Savary et al., 2019). While the situation seems dire, plant diseases are routinely managed using myriad techniques including but not limited to naturally occurring genetic plant resistance, fungicides and biocontrol agents. The first two strategies are effective, but simultaneously place selective pressures on the pathogen population to adapt. Evidence of pathogen evolution against plant Resistance (R) genes is demonstrated through studies on the rice blast fungus, *Magnaporthe oryzae*, via instability of their cognate avirulence proteins; these proteins are recognized by the plant’s R gene, but can accrue mutations, making it unrecognizable and subsequently undetected by the plant (reviewed in (Jia et al., 2016)). Fungicide usage, particularly when they target a single site in the pathogen, has led to development of fungicide resistance (reviewed in (Yin et al., 2023)). Again using *M. oryzae* as an example, strains of this pathogen have developed resistance to QoI fungicides, which target single sites to inhibit respiration in fungi (Avila-Adame and Köller, 2003; Castroagudín et al., 2015). Naturally occurring genetic resistance and fungicides result in a boom-and-bust cycle of resistance and disease, creating a need for alternative control measures.

Utilizing biological control agents to combat plant disease is predicated on the indirect or direct antagonistic interactions that these agents have against other microbes in their natural environment (reviewed in (Collinge et al., 2022)). Given their multiple modes of action, such as antibiosis, competition for resources and space, and enhancing plant growth, biocontrol agents show promise for avoiding these boom-and-bust cycles. Biological control agents include fungi such as *Trichoderma* spp., which are widely deployed worldwide (Lorito et al., 2010), and bacteria including but not limited to *Bacillus* species, which have many of the attributes listed above (reviewed in (Fira et al., 2018; Aloo et al., 2019). There have been 95 peer-reviewed publications in the last 20 years on potential biocontrol agents against the oomycete pathogen, *Phytophthora infestans*, causal agent of potato late blight (Hashemi et al., 2022). While most do not translate successfully to field applications, the mechanistic knowledge provides invaluable insight into stopping pathogens.

We previously isolated *Bacillus* (*B.*) *velezensis* strain S4 from biochar-amended agricultural soil in Delaware and sequenced its genome (Hempel et al., 2020). We hypothesized that this soil-borne strain of *B. velezensis* would inhibit the growth of foliar pathogens more than the aggressive, soil-borne plant pathogens *Rhizoctonia solani* and *Sclerotinia sclerotiorum*, which are likely to occupy the same niche, and to compete for the same resources (Boer et al., 2005). The prevalence of these two pathogens consistently causing plant diseases in Delaware indicates their success in competing with other microbes (Demissie et al., 2022; Ginn et al., 2023). Further, *B. velezensis* strains are emerging as promising biocontrol agents; Kjelgaard et al. demonstrated that a *B. velezensis* strain had potent anti-fungal activity in a high-throughput screen against the cereal pathogen, *Fusarium culmorum* (Kjeldgaard et al., 2022). In this study, we characterized strain S4 for putative enzyme activity, antimicrobial activity against five fungi and two oomycetes also isolated from Delaware soils, and evaluated its impact on spore germination of the rice blast fungus, *Magnaporthe oryzae*.

## 2 Materials and methods

### 2.1 Strains and growth conditions

#### 2.1.1 Fungal strains

*Magnaporthe oryzae* (strain 4091)*, Colletotrichum graminicola, Diaporthe ueckerae, Sclerotinia sclerotiorum, Rhizoctonia solani, Phytophthora nicotianae*, and *Pythium dissotocum* were grown on half-strength Potato Dextrose Agar (PDA; Becton, Dickinson and Company, MD, USA) in a 12:12 light:dark cycle incubator set at 25°C. *Magnaporthe oryzae* was grown for spore production on half-strength PDA, and subsequently filtered through miracloth (Sigma-Aldrich, MO, USA). All fungal and oomycete strains were isolated from Delaware soils (N. Gregory pers. comm.), except for *M. oryzae* strain 4091, which was a gift of J. Sweigard.

#### 2.1.2 Bacterial strains

*Bacillus velezensis* strain S4. *B. velezensis* strain S4 was isolated from agricultural soil as described previously (Hempel et al., 2020). For routine growth and transfer in the laboratory, it was grown in solid or liquid LB medium (Fisher Scientific, Pittsburgh, PA, USA) at 28°C. For long-term storage, a liquid culture was amended with glycerol at a final concentration of 10% and maintained at -80°C. Culture supernatant for challenging fungal cultures was prepared by transferring a single colony into LB and growing for 48 hours with shaking at ~30°C, 200 rpm. The culture supernatant was filtered through a 0.2 µm filter to remove whole cells.

*Bacillus velezensis* strain S10-2-W. This strain was isolated from the same soils as S4 and grown as per S4.

#### 2.1.3 Arabidopsis

Arabidopsis Col-0 seeds were obtained from the Arabidopsis Biological Resource Center (ABRC). Seed surfaces were decontaminated using 95% ethanol followed by 50% bleach, then rinsed 5X with sterile water. Sterilized seeds were plated on Murashige and Skoog (MS) medium (per liter, 4.4 g MS salt (BioWorld Plant Media, OH, USA), 10 g sucrose, 8 g agar; pH 5.7-5.8. Plates were incubated at 21 ± 2 °C with 12 h/12 h light/dark and illuminated with cool fluorescent light with an intensity of 120 μEm^-2^s^-1^ for 20 days.

### 2.2 Bacterial enzymatic plate assays

**Figure 1:**
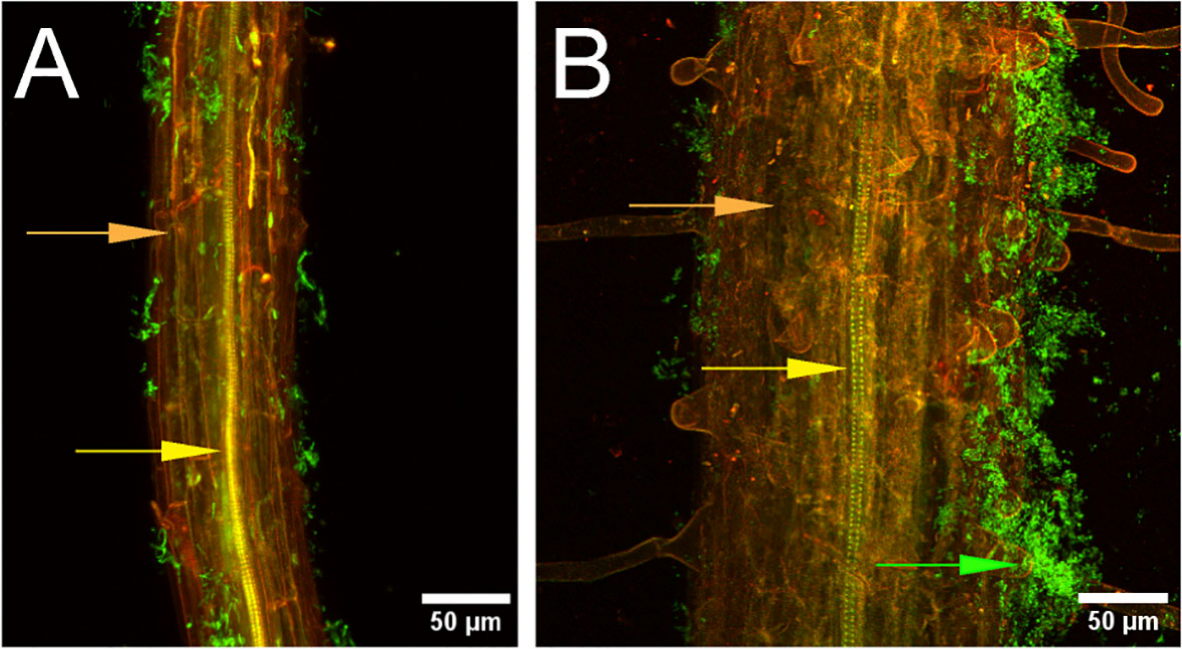
*Bacillus velezensis* strain S4 aggregates on *Arabidopsis thaliana* roots. Seedling roots were incubated with suspended *Bacillus velezensis* strains S10-2-W **(A)** or S4 **(B)** cells for 30 hours, then fixed, stained, and imaged. Green fluorescence indicates bacteria (green arrow) on root surface (orange). Xylem is indicated with the yellow arrow.

*Bacillus velezensis* strain S4 was grown in LB at 30°C for 16 hours at 80 rpm (OD_600 nm_= 0.5-0.7). Sterile filter papers (8 mm) were briefly dipped into overnight culture with sterile forceps. Each paper was placed in the middle of assay plates to test for enzymatic activity of proteases and lipase (**Figures S1A, B**); for cellulase activity, the filter paper was briefly dipped in LB or S4 overnight culture and pressed gently onto the center of their respective plates (**Figure S1C**). Protease activity was tested using skim milk agar (SMA), which includes 100 g skim milk powder (Bectin, Dickson and Co., Franklin Lakes NJ, USA) and 16 g agar per liter (**Figure 1**) (Baard et al., 2023). Lipase activity was tested using tributyrin agar (TA), which is composed of 12 g agar, 10 mL glyceryl tributyrate (Sigma-Aldrich, St. Louis MO, USA; protocol can be found here: https://www.sigmaaldrich.com/deepweb/assets/sigmaaldrich/product/documents/417/921/91015dat.pdf), 5 g peptone, 3 g yeast extract, and 6 drops of filter-sterilized 1:1 Tween 20: deionized water per liter (Sigma-Aldrich). Cellulase activity was tested using carboxymethylcellulose agar (CMC), which is composed of carboxymethylcellulose sodium salt 5 g (Sigma-Aldrich, St. Louis MO, USA), sodium nitrate 1 g, potassium chloride 1g, yeast extract 1 g, dipotassium phosphate 0.5 g, magnesium sulfate heptahydrate 0.5 g, and agar 15 g, pH 7.0., respectively (Batubara et al., 2021). After 72 hours, the cellulase plates were flooded with 0.1% Congo Red (10mL/each plate) and left at room temperature for 20 minutes, followed by washing with 1M NaCl (~30 mL). Parallel control plates were made using the same media and technique but using LB broth in place of overnight culture. All plates were incubated at 30°C. The protease and lipase plates observed for 3 days and cellulose plates for 2 days.

### 2.3 Bacterial genome analysis (anti-SMASH)

The genome of *B. velezensis* strain S4 was previously sequenced (Hempel et al., 2020). To identify gene clusters encoding the synthesis of potential antifungal molecules, the genome was analyzed using AntiSMASH v. 6.0.1 (Blin et al., 2019), with strict detection and default parameters for all other options.

### 2.4 Plant root association assays

*Bacillus velezensis* strains S4 and S10-2-W were revived from frozen stocks on solid LB medium and incubated at 28°C overnight. One colony was then transferred to liquid LB and incubated with shaking at 28°C for 12-18 hours, then harvested by centrifugation (4000 × g, 10 min) and washed with sterile water. The OD_600 nm_ was adjusted to 0.02 in 0.5X MS liquid medium (0.5 mM MES, 0.1% sucrose (Bais et al., 2004)).

Five, six-day old *Arabidopsis thaliana* Col-0 seedlings germinated on MS solid medium were transferred to a magenta box containing 20 mL 0.5X MS liquid medium without touching a mesh support. The mesh was provided to ensure that only roots would grow into the liquid medium. Plants were grown for 12 - 14 d with constant shaking at 90 rpm at 21 ± 2 °C with a 12 h/12 h photoperiod. After 12 d the medium was completely removed and replaced with a suspension of either *B. velezensis* strain S4 (OD_600 nm_ = 0.02) or S10-2-W in freshly prepared 0.5X MS liquid medium. The magenta boxes were shaken at 90 rpm for 24 h, then incubated for 6 h without shaking to promote biofilm formation (Bais et al., 2004). *A. thaliana* roots were then carefully removed from the magenta box, rinsed with PBS twice and fixed in 4% formaldehyde. The roots were stained with SYTO^®^13 (Invitrogen, Molecular Probes, OR, USA) for 5 min, then transferred to sterile water. Images were captured with a 10X Plan-Apochromat objective (numerical aperture 0.45) or 25X C-Apochromat objective (numerical aperture 1.2) on a Zeiss LSM 510 NLO attached to an Axiovert 200M with Zeiss AIM software (Rel. 3.2). Images were acquired with the 488 nm line excitation of an Argon laser using a 505 nm long-pass emission filter.

### 2.5 Diffusible molecule assay

*Bacillus velezensis* strain S4 was revived from glycerol stocks on solid LB and incubated overnight, then inoculated into 25 ml of liquid LB (Fisher Scientific, PA, USA) and incubated at 37°C with shaking at 80 rpm for 18 hours. Following the 18-hour incubation period, 500 μl of the overnight culture was transferred into 75 ml of liquid LB (Fisher Scientific, Pittsburgh, PA, USA), incubated at 37°C and 80 rpm for 6 hours, and either used immediately or stored at 10°C for several hours until used.

Assays used half-strength PDA in Fisherbrand 100 mm x 15 mm petri plates (Fisher Scientific, PA, USA). Fisherbrand Q5 9 cm filter paper units (Fisher Scientific, PA, USA) were hole-punched into 0.8 cm circles and autoclaved. On each half-strength PDA plate, one 0.8 cm sterile filter paper disk and one 0.8 cm fungal plug were placed 2 cm away from the edge of the petri plate, directly opposite of each other. 10 μl of the S4 bacterium overnight culture was then pipetted onto the center of the filter paper disk. Plates were sealed with 3M Micropore 1 inch tape (3M, St. Paul, MN, USA) and incubated at 25°C for 14 days. Various time points were used to record the radial measurements of the pathogen growth. Growth inhibition was calculated as [(*Rc*−*Rexp*)/*Rc*] ×100, where *Rc* represents the longest radius (in cm) of fungal mycelia measured from the center of the fungal plug, and *Rexp* is the shortest measured radius of fungal mycelia growing towards the bacterium (Chen et al., 2018b).

### 2.6 Recovery assay

Half-strength PDA plates were divided into four quadrants. Two sections of the pathogen (2 mm size cube) nearest to *B. velezensis* strain S4 colony on the diffusible molecule assay plates were cut out with sterile forceps and placed in the center of quadrants one and three (see **Figure S2A**). From the same plate, an additional two 2 mm cubes of the pathogen were cut from the region where the pathogen is furthest away from the bacteria and placed in the center of quadrants two and four. Two-millimeter cubes were also excised from the control plate (fungus or oomycete without S4) and transferred to half-strength PDA plate that was split into two quadrants. These two cubes were placed in opposing quadrants and monitored alongside the treatment plates for five days.

### 2.7 Microscopic observations of pathogen hyphae and spores

#### 2.7.1 Hyphal observation assays

Half-strength PDA plates were inoculated and incubated at 25°C with a 12h day/night cycle. Images were collected at 5, 6 and 7 days with a Zeiss Axiozoom V16 (Zeiss, Germany) using a Axiocam 512 color camera at 10, 25, 50 and 100x and analyzed using FIJI (Schindelin et al., 2012).

#### 2.7.2 Spore germination assays

We challenged *M. oryzae* spores with filtered supernatant from a *B. velezensis* strain S4 culture, grown to an OD_600 nm_ of 0.579. Fungal spores were isolated from half-strength PDA as described above and adjusted to a concentration of 6 x 10^5^ spores ml^-1^. Each well of a IBIDI u-bottom 96 well plate (IBIDI USA, Inc, Fitchburg, WI, USA) was inoculated with 100 μL of the spore suspension and 100 μL treatment: sterile water, nystatin (final concentration 20 mg mL^-1^ or 40 μg mL^-1^), or diluted, filtered supernatant from a 48 hour culture of *B. velezensis* strain S4 (1:1, 1:10 or 1:100 dilutions in sterile water). Each treatment was repeated in triplicate. The plate was incubated at 22°C for 2 hours. The wells were then imaged every hour for 16 hours on a CellDiscoverer7 (Zeiss, Germany). Data was acquired using a 20x plan-apochromat objective with a numerical aperture of 0.7. Post analysis acquisition was performed in Zeiss Zen Blue Lite 3.2 (Zeiss, Germany).

We also challenged *M. oryzae* spores directly with a live culture of *B. velezensis* strain S4. *B. velezensis* strain S4 was grown overnight as described above, then diluted 1:1 or 1:100 in sterile water. Each well of a 96-well plate was inoculated with 150 µl of *M. oryzae* spores (concentration of 5.9 x 10^5^ spores per mL) and 150 µl of sterile water or *B. velezensis* strain S4 culture (live 1:1, live 1:100, and undiluted, heat killed bacteria). Bacteria were heat-killed by incubating at 95°C for 30 min. Each treatment was repeated in triplicate. The 96-well plate was then incubated at room temperature overnight and placed into the CD7 for imaging. The germ tubes, appressoria, and ungerminated spores were counted manually using Zen 3.2. Three replicates of each treatment were counted with over 700 spores included in statistical analysis.

### 2.8 Statistical analyses

Statistical analysis was performed using the T-test function in Microsoft Excel comparing the effect of each treatment to the effect of water. Results were considered significant when the null hypothesis was rejected with p-values < 0.05.

## 3 Results

### 3.1 Bacterial genome analysis and characterization

*Bacillus velezensis* strain S4 was analyzed using the AntiSMASH tool (Blin et al., 2019), which identified 12 gene clusters putatively encoding synthesis of antimicrobial or antifungal compounds (**Table 1**), including clusters for synthesis of bacillaene, bacillibactin, bacilysin, difficidin, fengycin, macrolactin, and mersacidin (all with 100% identity to known gene clusters in *Bacillus* spp.); plantazolicin and surfactin (91% identity to known gene clusters in *Bacillus* spp.); and two putative terpenes and a Type III polyketide synthase (T3PKS) with low identity to known gene clusters in other groups of bacteria. Surfactin, bacillaene, fengycin, bacillibactin, and bacilysin have been shown to have antifungal activity in prior work (Koumoutsi et al., 2004; Chen et al., 2009; Um et al., 2013; Hazarika et al., 2019; Dimopoulou et al., 2021). Strain S4 was then assayed on solid media containing different substrates to assess extracellular enzyme activity. The bacterium produced a large clear zone around the inoculation point on skim milk agar, and a small halo on carboxymethylcellulose agar indicating putative protease and cellulose activity, respectively (**Figure S1**) but suggests a lack of lipase activity under our conditions. While S4 grew on tributyrin agar, there was no zone of clearing observable. The *B. velezensis* strain S4 genome has 42 annotated proteases, 7 annotated lipases, and 7 annotated enzymes putatively involved in cellulose degradation, including endoglucanase, β-glucosidase, β-glucanase and exoglucanase enzymes (Chen et al., 2018a; Singh et al., 2019; Hempel et al., 2020).

**Table 1:** Predicted secondary metabolites with antifungal or antibacterial activity identified in the genome of *B. velezensis* strain S4.

Product	Genome location	Closest match in NCBI (accession no.)	% ID^1^	Closest match in AntiSMASH	% Sim^2^
Bacillaene^3^	1,746,698- 1,847,275	*B. velezensis* strain FLU-1 (CP125279.1)	99.4%	*B. velezensis* strain FZB42 (AJ575642.1)	100%
Bacillibactin^3^	3,124,828- 3,176,621	*B. velezensis* strain AS43.3 (CP003838.1)	99.4%	*B. subtilis* subsp. *subtilis* str. 168	100%
Bacilysin^3^	3,702,102- 3,743,520	*B. velezensis* strain FLU-1 (CP125279.1)	99.9%	*B. velezensis* strain FZB42 (AJ575642.1)	100%
Difficidin	2,374,517- 2,468,311	*B. velezensis* strain FLU-1 (CP125279.1)	99.6%	*B. velezensis* strain FZB42 (AJ575642.1)	100%
Fengycin^3^	1,917,959- 2,053,491	*B. velezensis* strain HF14109 (CP103412.1)	99.5%	*B. velezensis* strain FZB42 (AJ575642.1)	100%
Macrolactin	1,439,335- 1,527,184	*B. velezensis* strain AS43.3 (CP003838.1)	99.4%	*B. velezensis* strain FZB42 (AJ575642.1)	100%
Mersacidin	3,900,122- 3,923,310	*B. velezensis* strain KS04AU (CP092750.1)	99.7%	*Bacillus* sp. HIL-Y85/54728	100%
Plantazolicin	739,670- 761,852	*B. velezensis* strain SRCM102747 (CP028211.1)	99.1%	*B. velezensis* strain FZB42 (AJ575642.1)	91%
Surfactin^3^	313,657- 378,516	*B. velezensis* strain FLU-1 (CP125279.1)	99.6%	*B. velezensis* strain FZB42 (AJ575642.1)	91%
Type III polyketide synthase	2,204,160- 2,245,260	*B. velezensis* strain AS43.3 (CP003838.1)	99.7%	Myxobacteria, Streptomyces	41-43%
Terpene	1,106,948- 1,124,178	*B. velezensis* strain AS43.3 (CP003838.1)	99.5%	Streptomyces, Myxobacteria	41-42%
Terpene	2,118,863- 2,245,260	*B. velezensis* strain TrigoCor1448 (CP007244.1)	99.4%	Streptomyces	56%

^1^indicates nucleotide identity to the closest match in the NCBI non-redundant database, as determined by blastn.

^2^was calculated by AntiSMASH and indicates the percentage of genes in the homologous gene cluster in the closest match that have a significant blast hit to genes in the query genome (here, B. velezensis strain S4).

^3^indicates compounds with documented antifungal activity.

AntiSMASH was used to identify putative biosynthetic gene clusters for antifungal or antibacterial products. The majority are very similar or identical to gene clusters found in other *B. velezensis* strains.

### 3.2 *B. velezensis* strain S4 associates with plant roots

Root assays were performed on Arabidopsis ecotype Col-0 to determine whether strain S4 showed aggregation on root surfaces, potentially indicative of a biofilm formation. It was compared with strain S10-2-W, which was isolated from the same soil as S4, but showed moderate antifungal activity (S.M. Yannarell and J. Maresca, pers. comm.). Results demonstrated that while some bacterial cells from the S10-2-W strain were scattered on the root surface, S4 formed large aggregates. Optical slices (z-stacks) were taken through the root inoculated with S4 and no bacteria were visualized inside the root, indicating they are not entering (data not shown).

### 3.3 Diffusible molecule assays

#### 3.3.1 Effect of B. velezensis strain S4 on pathogen growth

**Figure 2:**
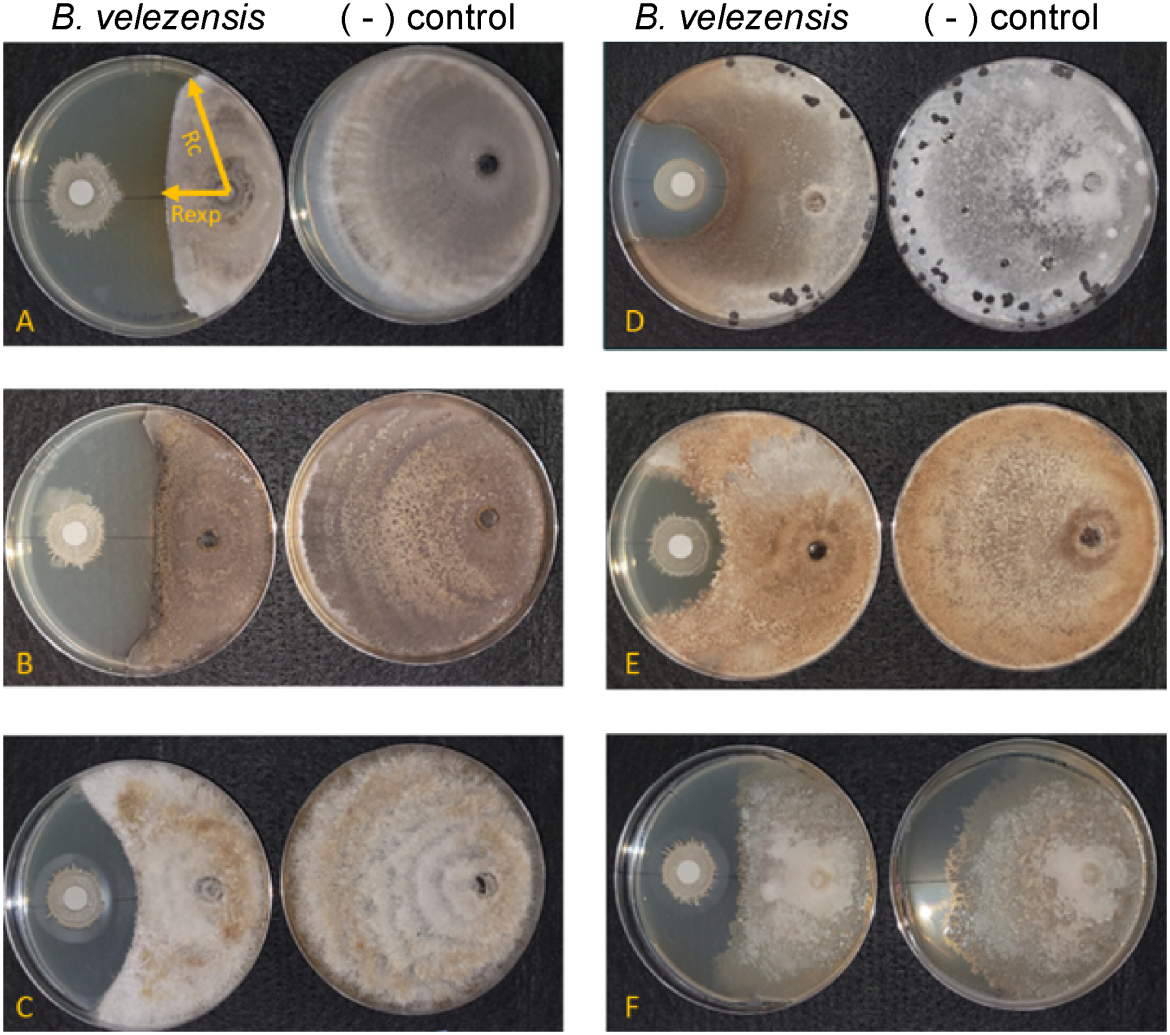
*Bacillus velezensis* S4 inhibits growth of five fungi and one oomycete. **(A)** *Magnaporthe oryzae*; **(B)** *Colletotrichum graminicola*; **(C)** *Diaporthe ueckerae*; **(D)** *Sclerotinia sclerotiorum*; **(E)** *Rhizoctonia solani*; **(F)** *Phytophthora nicotianae*. For each panel, the left plate is the diffusible molecule assay with the bacterial strain spotted onto a sterile filter paper and the right plate is the fungus growing in the absence of the bacterial cells. Images are representative of six biological replicates.

We used *in vitro* antifungal assays to determine whether strain S4 had inhibitory properties against fungi and oomycetes. Previous studies have shown that inhibitory compounds can diffuse through the media, creating a flattened, or inhibition zone, of the fungal colony (Anith et al., 2021; Kjeldgaard et al., 2022). We have access to a collection of soilborne and foliar fungal and oomycete plant pathogens, and except for *M. oryzae* strain 4091, all were isolated from Delaware plant and soil samples (N. Gregory, pers. comm.). We measured fungal growth along the radius from the center of the fungal plug to the center of the bacterial filter paper, along with the longest radius of the fungal colony. Assays were performed on five fungi and two oomycetes. While growth of all the fungi tested and one oomycete (*P. nicotianae*) tested was significantly inhibited when compared to the pathogen growing in absence of the bacterium (**Figure 2**), *D. ueckerae* and *C. graminicola* showed the most susceptibility to the diffusible molecules (**Table 2**). The growth of the water- and soilborne oomycete, *P. dissotocum*, was not significantly inhibited; however, the hyphae were observably less dense over the bacterial colony than in the rest of the colony or the control (**Figure S3**).

**Table 2:** *B. velezensis strain S4 significantly inhibits growth of 5 fungi and 1 oomycete*.

	Soil or foliar	Pathogen name	% growth inhibition^a^
Fungi	*Foliar*	*Colletotrichum graminicola*	48.44 ± 1.57*
	*To be determined*	*Diaporthe ueckerae*	48.28 ± 1.69*
	*Foliar*	*Magnaporthe oryzae* strain 4091^b^	38.53 ± 5.76*
	*Soil*	*Rhizoctonia solani*	30.31 ± 3.46*
	*Soil*	*Sclerotinia sclerotiorum*	39.94 ± 1.48*
Oomy-cetes	*Both*	*Phytophthora nicotianae*	29.89 ± 3.45*
	*Soil*	*Pythium dissotocum*	3.03 ± 0

a Growth inhibition was calculated using [(*Rc*-*Rexp*)/*Rc*] ×100, where *Rc* represents the longest radius (in mm) of fungal mycelia measured from the center of the fungal plug, and *Rexp* is the shortest measured radius of fungal mycelia growing towards the bacterium.

b Growth of M. oryzae on plates was inconsistent, so fewer replicates were performed.

*indicates p < 0.05.

Inhibition of pathogen growth by *B. velezensis* strain S4 ranged from 30% to nearly 50% as compared to water. Each value is the average and standard deviation of 6 replicates. Significance was determined using a student’s T-test; results were considered significant if the p-value was less than 0.05.

We then performed recovery assays on fungi and oomycetes from the diffusible molecule assays to determine whether the inhibitory metabolites from strain S4 had fungistatic or fungicidal effects. Two sections from the zone of inhibition, two from the colony not directly in the path of the molecules, and two from the control plates, were plated onto fresh media, grown for five days and imaged (**Figure S3**). Except for *C. graminicola* and *S. sclerotiorum*, strain S4 was clearly present when extracting fungi from the inhibition zone, as it grew out on the quadrant plates and continued to inhibit the fungal colonies. All fungi and oomycetes grew out on recovery plates, indicating that fungal cells are inhibited by exudates secreted by S4, but might not be killed. Alternately, we might have excised some live cells when transferring to the recovery plates.

#### 3.3.2 Diffusible molecules from the bacterium inhibit normal hyphal growth and spore germination

**Figure 3:**
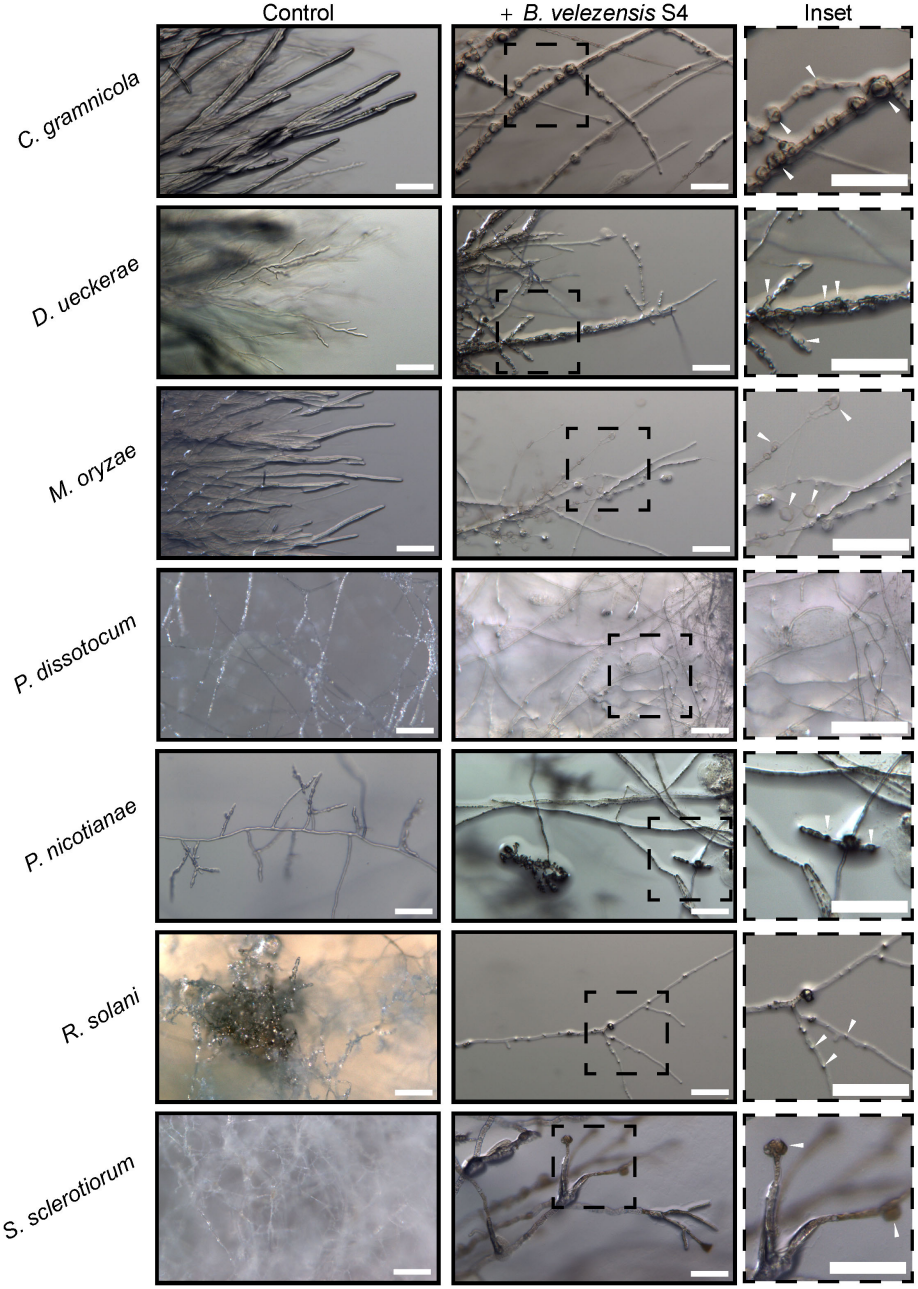
Five fungi and two oomycetes were subjected to *Bacillus velezensis* S4 small molecules using the diffusible molecule plate assay. Seven days post inoculation, the inhibition zone between microbes was imaged for structural changes at the macroscopic level using a Zeiss Axio Zoom dissecting microscope.

To begin understanding the mechanism of biocontrol, we first used a dissecting microscope to examine the hyphae in the zone of inhibition for macroscopic changes in structure. In all the fungi, hyphae formed bubble-like structures in the inhibition zone (**Figure 3** inset), similar to work from He et al (He et al., 2019) with *M. oryzae*. No bubbles formed in the hyphae of the oomycete pathogens *P. nicotianae* and *P. dissotocum*, however we observed less branching of the hyphae, particularly in *P. nicotianae*, and some thickened structures that were difficult to identify (**Figure 3**). The *Pythium* colony grew over the S4 bacterial colony in the diffusible molecule assay, so growth was not inhibited; however, the hyphae directly over the colony were much less dense than on the rest of the plate, or on the control (**Figure S3**). Interestingly, we also noted that the *S. sclerotiorum* colony, usually white except for the sclerotial bodies, were highly pigmented at the zone of inhibition (**Figure 2D**). In this zone, the hyphae were no longer hyaline, and hyphal tips seemed to terminate in bubble-like structures.

**Figure 4:**
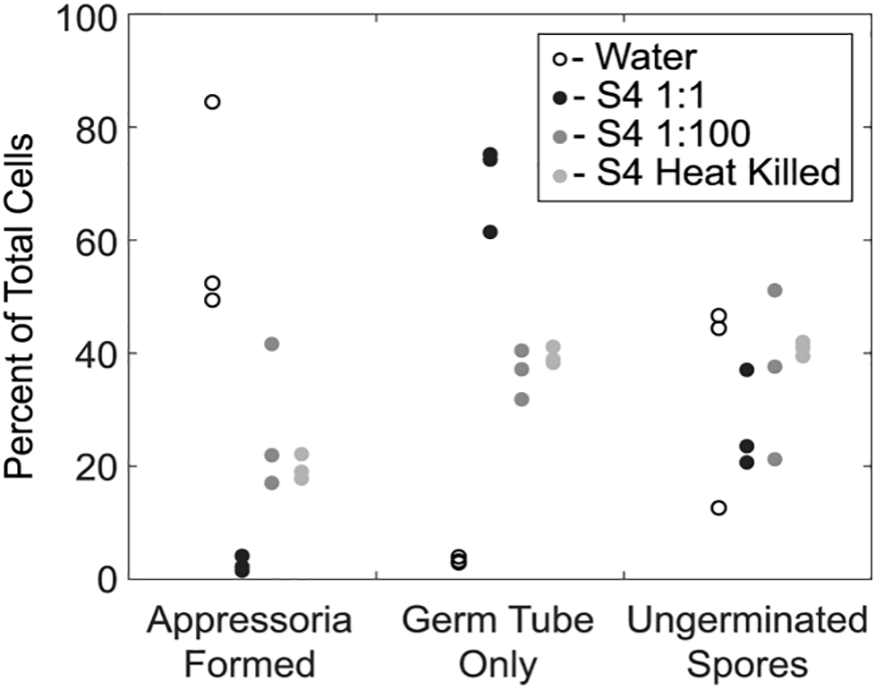
Germination of *Magnaporthe oryzae* in the presence of S4 shows reduction of appressoria formation. An overnight culture of *Bacillus velezensis* S4 was added to *M. oryzae* conidia and examined for differences in germination and appressorial formation. The bacterial culture was added to conidia in a 1:1 and 1:100 dilutions, and as a heat-killed culture. * p-value <0.05, which was considered statistically significant in a one-tailed student’s t-test.

We next examined the impact of strain S4 diffusible molecules on asexual growth of *M. oryzae* spores. Infection biology of this pathogen is well-studied and begins with an asexual spore, or conidium, landing on an inductive, hydrophobic surface (Choi and Dean, 1997). The conidium then germinates and forms a penetration structure, or appressorium, leading to penetration of the epidermal cells of the plant (Howard et al., 1991). We incubated *M. oryzae* conidia with water, two dilutions of *B. velezensis* strain S4, or a heat-killed overnight culture of the bacteria for 20 hours, then imaged germ tube and appressoria formation. Under typical *in vitro* conditions, a spore will germinate and form a melanized, bulbous appressorium in 9 to 12 hours. Here, the number of ungerminated spores in each treatment was the same. In wells treated only with water, most spores that germinated also formed appressoria, so few conidia with only germ tubes were observed (**Figure 4**). In the presence of the highest concentration of the bacterium (1:1 dilution), many germ tubes developed but few appressoria formed (**Figure 4**). More appressoria formed in the wells treated with 1:100 diluted culture of *B. velezensis* strain S4, with a concomitant reduction in cells with germ tube formation only (**Figure 4**). Treatment with the heat-killed bacterial culture was not significantly different from the 1:100 dilution.

**Figure 5:**
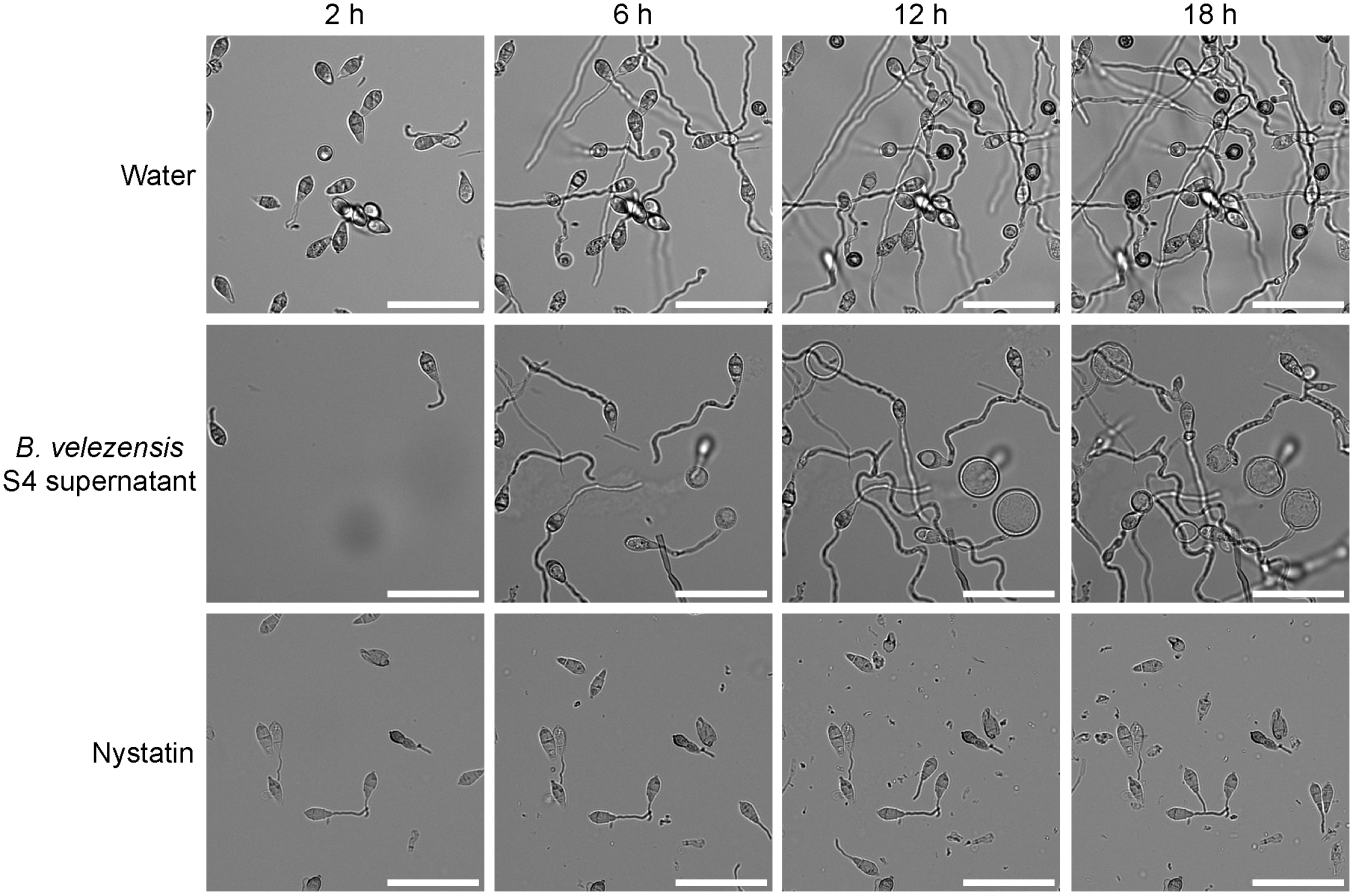
S4 supernatant deforms germinating *Magnaporthe oryzae* spores. Filtered supernatant from a *Bacillus velezensis* strain S4 culture was applied to isolated *M. oryzae* spores and the spores were imaged over time. Appressoria typically begin forming around 6 hours and become fully melanized at 12 hours. In the presence of the S4 supernatant, bubbles appear to differentiate from the germinating spores and no appressoria were formed. Nystatin was used as a germination control. Scale bar equals 50 μm.

The effects of supernatant from a *B. velezensis* strain S4 culture (**Figure 5**) were similar to the effects of diluted live *B. velezensis* (**Figure 4**) on *M. oryzae* spore germination and appressorial formation. Four time points during germination on an inductive surface were examined and revealed bubbles forming at 6 and 12 hours, instead of appressoria (**Figure 5**). Nystatin, which inhibits appressorium formation but not germination (Eilbert et al., 1999) was used as a control. Together, our observations indicate that the diffusible molecules from strain S4 disrupt normal vegetative growth and conidial germination of *M. oryzae*, forming bubble-like swellings instead of typical penetration structures on an inductive surface.

## 4 Discussion

We isolated a potential biocontrol bacterium from biochar-amended agricultural soils in Delaware. *Bacillus velezensis* strain S4 displays potent inhibition of five plant pathogenic fungi, as well as one plant pathogenic oomycete. We predicted that this strain would minimally inhibit growth of soil-borne pathogenic fungi. However, it significantly reduced the growth of *Rhizoctonia solani* and *Sclerotinia sclerotiorum*, both found in soil, and was even more effective at inhibiting the foliar fungi *C. graminicola* and *M. oryzae* as well as the oomycete *P. nicotinianae. Diaporthe ueckerae* is an emerging pathogen of particular concern on soybeans, as it is one of several species causing seed decay, however this species has not yet been characterized as foliar or soil-borne (Petrović et al., 2021; A. Koehler, pers. comm.). This specific isolate has not yet been characterized as foliar or soil borne (A. Koehler, pers. comm.). Growth of the soil-borne oomycete *Pythium dissotocum* was less affected; however, its hyphae did become sparse as it grew over the bacterial colony.

Our results demonstrated strong inhibition of the foliar pathogens *C. graminicola* and *M. oryzae*, and weaker but significant inhibition of the aggressive, soilborne pathogens *S. sclerotiorum* and *R. solani*. We observed that *S. sclerotiorum* hyphae, usually hyaline except for its survival structures (sclerotia), was melanized at the inhibition zone and displayed thickened hyphae, many of them terminating in bubble-like structures. The melanin biosynthetic pathway is involved in producing sclerotial bodies (Butler et al., 2009), although one recent study indicates that when the major genes for melanin production are deleted from this fungus, it grows more slowly, is less resistant to UV light, but remains fully pathogenic (Liang et al., 2018). Melanization in the zone of interaction between *S. sclerotiorum and B. velezensis* suggests that melanin may also play a role in protecting *S. sclerotiorum* from antagonists. Song et al. isolated a *B. velezensis* strain from farm soils in South Korea that demonstrated varying levels of inhibition against two different species of *S. sclerotiorum* and *Colletotrichum* (Song et al., 2022). The former was melanized, but it is unclear whether their *S. sclerotiorum* strain has non-pigmented hyphae under normal conditions. *S. sclerotiorum* is a broad-range, soilborne pathogen known to infect 425 plant species, but likely infects many more (Derbyshire et al., 2022). It has been difficult to manage for many reasons: infection with *S. sclerotiorum* is unrelated to crop loss in the field, and the environmental variables that impact life stage of *S. sclerotiorum* in the lab are not relevant in the field (reviewed in (Reich and Chatterton, 2022)).

*Rhizoctonia solani* is another broad host range, soilborne pathogen. We noted clear inhibition of the pathogen by *B. velezensis* strain S4, as well as hyphae forming into bubble-like structures. This differs from a recent study deploying *B. subtilis* strain SL-44 against a *R. solani* isolate responsible for large losses to pepper plants. Like *B. velezensis* strain S4, the *B. subtilis* SL-44 encodes the biosynthesis of fengycin, a noted antifungal compound. Unlike *B. velezensis* strain S4, their microscopic observations revealed hyphal breakage in *R. solani* instead of bubbles, with loss of cytosol and cell wall integrity (Wu et al., 2019). A more recent study used a different strain of *B. velezensis* against multiple isolates of *R. solani* causing brown patch on turf, and demonstrated that globoid, rounded structures formed at the tips of the hyphae in the inhibition zone (Lee et al., 2023). In contrast, we observed bubble formation behind the hyphal tip, potentially indicating that different strains of *B. velezensis* will produce different effects on the pathogens, though all may result in biocontrol.

The bubble-like structures we observed in the inhibition zones of all fungi tested, are also consistent with the results of a recent study on biocontrol of *M. oryzae* (He et al., 2019). There, *B. subtilis* strain BJ-1 was tested as a biocontrol agent against *M. oryzae*. The bacteria did not inhibit germination, but did disrupt the cell walls of the hyphae, as well as appressorial development. This is supported by the presence of genes for fengycin production, which has been reported to disrupt hyphal membranes, eventually leading to cell death (Xiao et al., 2021). Furthermore, *B. velezensis* strain S4 contains the synthetic machinery for bacilysin production, which is known to target cell wall biosynthesis (reviewed in (Andrić et al., 2020). Together, these compounds could account for the bubble-like structures formed within the hyphae, and at the terminal branches of the hyphae in the case of *S. sclerotiorum*. Indeed, Xiao et al. demonstrated that when the take-all pathogen of wheat was challenged with fengycin, hyphae formed circular structures at the tip (Xiao et al., 2021).

We performed recovery assays from sections of hyphae in the zone of inhibition and found that the fungi recover on fresh media, possibly indicating that strain S4 has a fungistatic rather than a fungicidal activity. However, further studies are needed either that the diffusing molecule(s) do not kill the pathogens, or we are collecting pathogen cells that are not in contact with the S4 exudates. While our search was not exhaustive, we were unable to identify recovery assays in the *B. velezensis* literature. While these results indicate that strain S4 is fungistatic, future studies will reveal whether fungal and oomycete cells in the inhibition zone are dead or just deformed, but still alive and able to recover.

There has been a recent surge in interest in *B. velezensis* strains for biocontrol, with several demonstrating both antifungal and plant growth promoting activities. A recent study on the *B. velezensis* strain FKM10, isolated from an apple orchard in China, suggests that it inhibits apple pathogens, and increases traits like height and fresh weight (Wang et al., 2020). A study from Shin et al. (2021) isolated and characterized a *B. velezensis* strain from pepper fields in South Korea, which indicate both fungal inhibition and plant growth promoting properties. However, neither study assessed aggregation of *B. velezensis* on plant roots. Here, we used microscopy to discern whether strain S4 was at least forming aggregates on roots, as other *B. subtilis* strains with known plant growth promoting and root binding properties do (Rudrappa et al., 2008). We observed large aggregates of S4 on Arabidopsis roots compared with another *Bacillus* strain that had moderate levels of antifungal activity. We did not observe the bacterium entering plant roots, and further studies are required to determine whether they are forming biofilms and/or whether they have plant growth promoting properties.

Genomic studies of strain S4 reveal an enrichment in potential antibiotic clusters, five of which likely have antifungal activity: surfactin, bacillaene, fengycin, bacillibactin, and bacilysin have been shown to have antifungal activity in prior work (Koumoutsi et al., 2004; Chen et al., 2009; Um et al., 2013; Hazarika et al., 2019; Dimopoulou et al., 2021). We further observed protease and cellulase activity in plate assays, which corresponded with putative protease and cellulose-degrading genes found in the genome. The bioactive compounds could be extracellular enzymes, secondary metabolites, or both, and the *B. velezensis* strain S4 genome is predicted to encode the synthesis of five small molecules with antifungal activity as well as enzymes capable of protein, triglycerides and cellulose degradation.

Biocontrol has tremendous potential to augment more traditional means of plant disease control, such as fungicide application and genetic resistance. Since biocontrol agents tend to have multiple methods of inhibiting or killing the pathogen, as many strains of *B. velezensis* do (Chen et al., 2018a; Borriss et al., 2019; Rabbee et al., 2019), evolving genetic resistance to biocontrol is not as straightforward as it would be against a fungicide with a single mode of action. Bardin and Nicot asked whether it is possible for fungi and oomycetes to develop resistance to biocontrol agents (Bardin et al., 2015). The answer will require fully understanding both the mechanisms of inhibition deployed by the biocontrol agent and the responses of the plant pathogens. Changes in hyphal morphology have been demonstrated by multiple labs, including ours, and the next stage will be to examine the genetics of pathogen response, whether the pathogen signals to itself when under duress, and ultra-structural changes within the pathogen cells at the inhibition zone.

## Data availability statement

The raw data supporting the conclusions of this article will be made available by the authors, without undue reservation.

## Author contributions

AW: Investigation, Methodology, Validation, Writing – original draft, Writing – review & editing. KC: Conceptualization, Investigation, Methodology, Writing – original draft, Writing – review & editing. JC: Formal analysis, Methodology, Visualization, Writing – review & editing. TC: Conceptualization, Investigation, Methodology, Visualization, Writing – review & editing. MM: Conceptualization, Data curation, Methodology, Writing – review & editing. SY: Investigation, Methodology, Visualization, Writing – review & editing. JM: Conceptualization, Data curation, Formal analysis, Investigation, Methodology, Supervision, Visualization, Writing – original draft, Writing – review & editing. ND: Conceptualization, Formal analysis, Funding acquisition, Investigation, Methodology, Project administration, Supervision, Writing – original draft, Writing – review & editing.

## Supplementary material

Supplementary Figure 1
*B. velezensis strain S4 shows protease and cellulose activity*. **(A)** skim milk agar; **(B)** carboxymethylcellulose agar with Congo red staining (light orange around the bacterial colony indicates halo); **(C)** tributyrin agar. For **(A, B)**, filter papers were inoculated with LB as the control (left plates) and with S4 (right plates) and incubated for 72 hours. For **(C)**, filter papers were dipped in LB control (left plates) or S4 (right plates) and stamped briefly onto the plate, lifted off, and incubated for 48 hours.

Supplementary Figure 2 Recovery assays show differing amount of hyphal growth. **(A)** Diagram of recovery assays. Quadrants 2 and 4 are isolated from the side of the plate furthest from the bacterial filter paper, while 1 and 3 are isolated from the zone of inhibition, closest to the bacterial filter paper. For B – G, the left plate shows hyphae isolated from control plates in the absence of S4 and the right plate shows hyphae isolated from antifungal assays. Images were taken 5 days post-inoculation. **(B)** *M. oryzae;* **(C)** *C. graminicola;* **(D)** *D. uekerae;* **(E)** *R. solani;* **(F)** *S. sclerotiorum;* **(G)** *P. nicotianae*

Supplementary Figure 3
*Pythium dissotocum* outgrows the bacterial strain. **(A)** *P. dissotocum* seven days after plating. The left plate has the *B. velezensis* S4 bacterial culture on the filter paper, while the right plate only has *P. dissotocum*. **(B)** Recovery assay. In quadrants 1 and 3, taken from the inhibition zone (less dense hyphae), bacterial growth is clear, however the oomycete appears largely unaffected, except for some thinner hyphae around the S4 colony.
