# Supplementary figures and images for: A Bacillus velezensis strain shows antimicrobial activity against soilborne and foliar fungi and oomycetes
Source: Front Fungal Biol. 2024 Feb 23;5:1332755. doi: 10.3389/ffunb.2024.1332755 (PMC10920214; doi:10.3389/ffunb.2024.1332755)

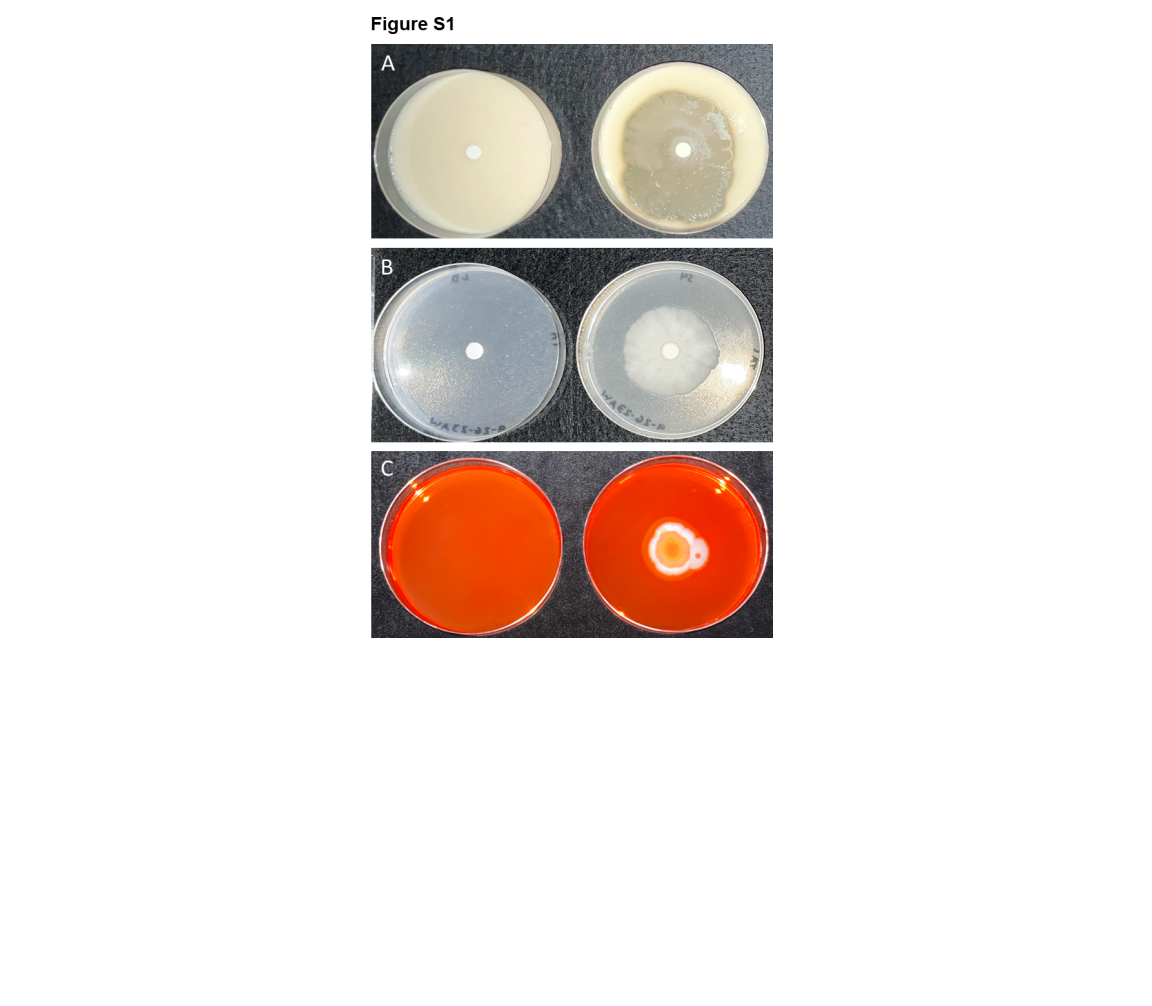

Supplement: Supplementary Figure 1 — B. velezensis strain S4 shows protease and cellulose activity. (A) skim milk agar; (B) carboxymethylcellulose agar with Congo red staining (light orange around the bacterial colony indicates halo); (C) tributyrin agar. For (A, B), filter papers were inoculated with LB as the control (left plates) and with S4 (right plates) and incubated for 72 hours. For (C), filter papers were dipped in LB control (left plates) or S4 (right plates) and stamped briefly onto the plate, lifted off, and incubated for 48 hours. [file Image_1.tif]

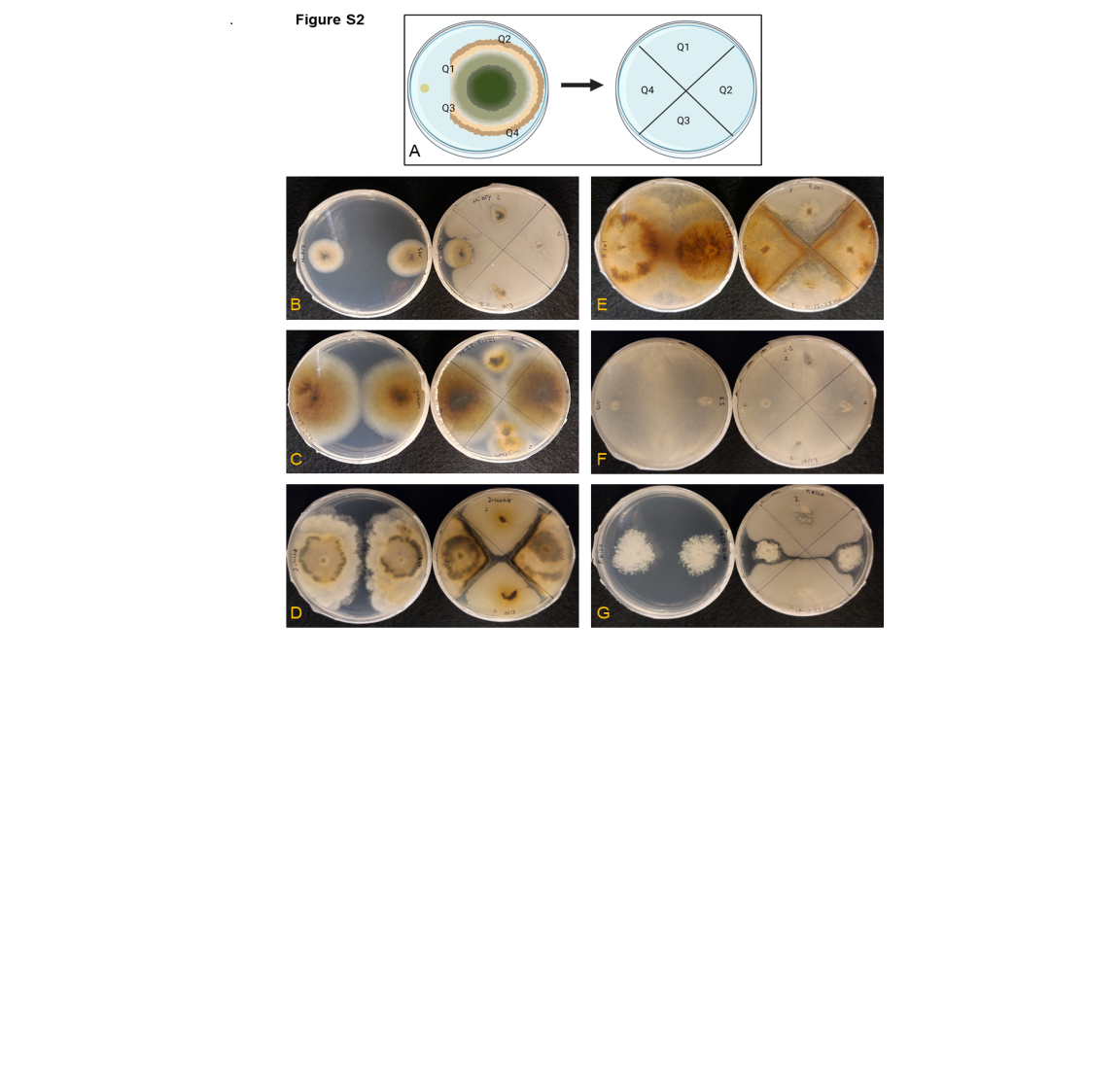

Supplement: Supplementary Figure 2 — Recovery assays show differing amount of hyphal growth. (A) Diagram of recovery assays. Quadrants 2 and 4 are isolated from the side of the plate furthest from the bacterial filter paper, while 1 and 3 are isolated from the zone of inhibition, closest to the bacterial filter paper. For B – G, the left plate shows hyphae isolated from control plates in the absence of S4 and the right plate shows hyphae isolated from antifungal assays. Images were taken 5 days post-inoculation. (B) M. oryzae; (C) C. graminicola; (D) D. uekerae; (E) R. solani; (F) S. sclerotiorum; (G) P. nicotianae [file Image_2.tif]

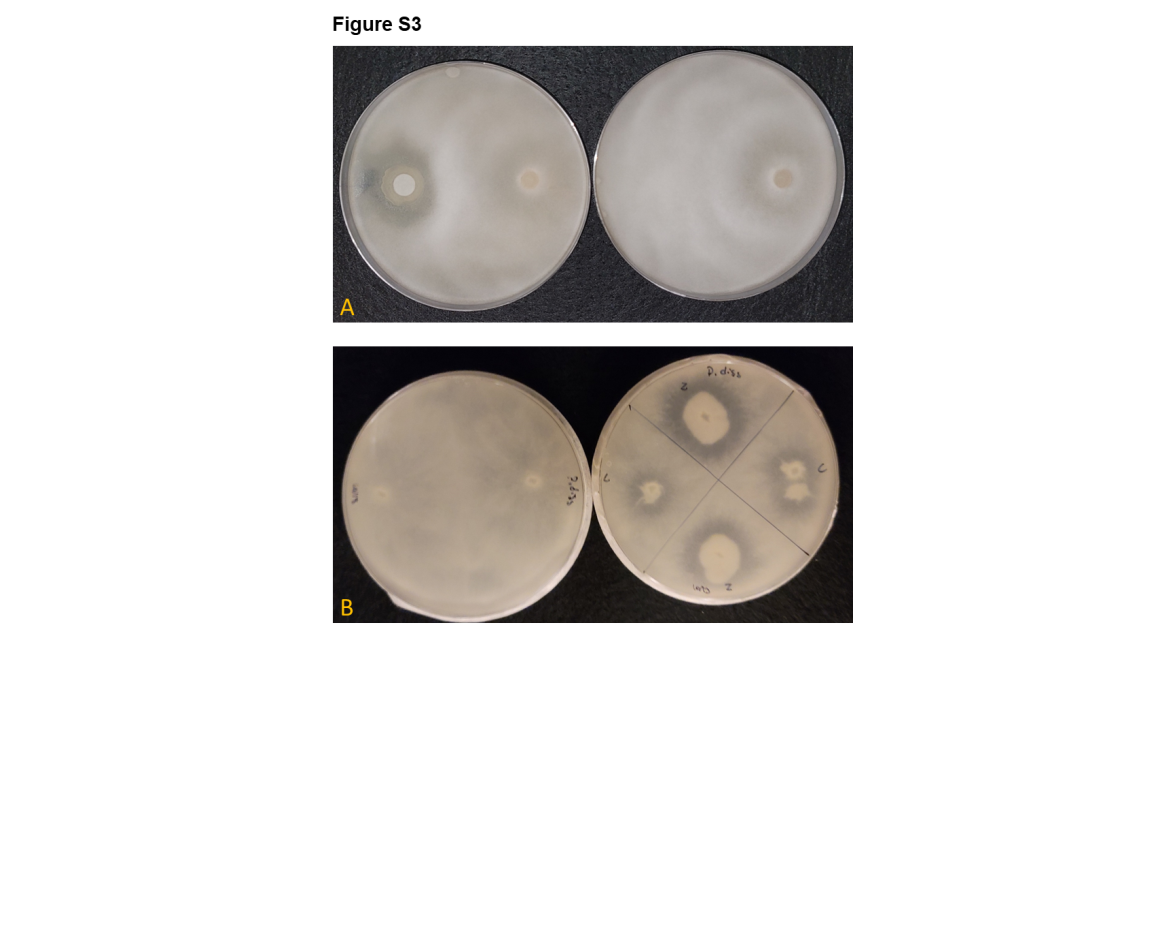

Supplement: Supplementary Figure 3 — Pythium dissotocum outgrows the bacterial strain. (A) P. dissotocum seven days after plating. The left plate has the B. velezensis S4 bacterial culture on the filter paper, while the right plate only has P. dissotocum. (B) Recovery assay. In quadrants 1 and 3, taken from the inhibition zone (less dense hyphae), bacterial growth is clear, however the oomycete appears largely unaffected, except for some thinner hyphae around the S4 colony. [file Image_3.tif]
